# Opiate Drugs with Abuse Liability Hijack the Endogenous Opioid System to Disrupt Neuronal and Glial Maturation in the Central Nervous System

**DOI:** 10.3389/fped.2017.00294

**Published:** 2018-01-23

**Authors:** Kurt F. Hauser, Pamela E. Knapp

**Affiliations:** ^1^Department of Pharmacology and Toxicology, Virginia Commonwealth University School of Medicine, Richmond, VA, United States; ^2^Department of Anatomy and Neurobiology, Virginia Commonwealth University School of Medicine, Richmond, VA, United States; ^3^Institute for Drug and Alcohol Studies, Virginia Commonwealth University School of Medicine, Richmond, VA, United States

**Keywords:** opioid drug abuse, glial maturation, neuronal maturation, perinatal development, human immunodeficiency virus, pediatric acquired immunodeficiency syndrome, neural stem cells, fetal abstinence syndrome

## Abstract

The endogenous opioid system, comprised of multiple opioid neuropeptide and receptor gene families, is highly expressed by developing neural cells and can significantly influence neuronal and glial maturation. In many central nervous system (CNS) regions, the expression of opioid peptides and receptors occurs only transiently during development, effectively disappearing with subsequent maturation only to reemerge under pathologic conditions, such as with inflammation or injury. Opiate drugs with abuse liability act to modify growth and development by mimicking the actions of endogenous opioids. Although typically mediated by μ-opioid receptors, opiate drugs can also act through δ- and κ-opioid receptors to modulate growth in a cell-type, region-specific, and developmentally regulated manner. Opioids act as biological response modifiers and their actions are highly contextual, plastic, modifiable, and influenced by other physiological processes or pathophysiological conditions, such as neuro-acquired immunodeficiency syndrome. To date, most studies have considered the acute effects of opiates on cellular maturation. For example, activating opioid receptors typically results in acute growth inhibition in both neurons and glia. However, with sustained opioid exposure, compensatory factors become operative, a concept that has been largely overlooked during CNS maturation. Accordingly, this article surveys prior studies on the effects of opiates on CNS maturation, and also suggests new directions for future research in this area. Identifying the cellular and molecular mechanisms underlying the adaptive responses to chronic opiate exposure (e.g., tolerance) during maturation is crucial toward understanding the consequences of perinatal opiate exposure on the CNS.

## Introduction

Maternal opiate abuse and neonatal abstinence syndrome are increasing at an alarming rate and this is in large part fueled by increases in the illicit use of prescription opiates (1–4). A 2015 press release from the National Institute on Drug Abuse noted that “Delivering mothers using or dependent on opiates rose nearly fivefold from 2000 to 2009” (5). Opiate drugs with abuse liability alter brain development through direct and indirect actions on neuronal and glial maturation. The goal of this review is to examine our current understanding of the direct cellular and molecular effects of opiates on central nervous system (CNS) maturation. Although it was well established in the 1970s that *in utero* and perinatal exposure to opiate drugs would hinder brain maturation, not until the early 1990s was it realized that opiates *per se*, through direct actions on opioid receptor-expressing immature neurons and glia, could intrinsically affect the maturation of the CNS. This realization by no means discounts or understates the importance of the myriad psychosocial and psychiatric (e.g., anxiety and depression) problems, comorbid bacterial (e.g., pneumonia and sexually transmitted diseases) and viral [e.g., human immunodeficiency virus (HIV)] infections (bacterial and viral), endocrine (6), pulmonary, and cardiovascular complications (7, 8) resulting from opiate abuse that also influence brain development. Rather, by elucidating the direct developmental consequences of opiate exposure that are unavoidable even, if all the other comorbid psychosocial and medical problems associated with opiate addiction are treated, should provide insight into how to better manage the CNS consequences of perinatal opiate exposure and neonatal abstinence syndrome. Accordingly, a major goal of our studies has been to determine the direct cellular consequences of opioid exposure on brain maturation in children; rather than to study addiction *per se*. Many of the brain regions that are most dramatically affected by perinatal opioids are thought to be unrelated to addiction. Although understanding the underlying neural substrates of addiction is a critically important problem, and some speculative discussion on how developmental exposure to opioids might contribute to addiction in adults is provided in this review, many of the effects of opioid exposure presumably unrelated to addiction are essential for understanding the pathophysiological consequences of perinatal opioid exposure. Finally, the clinical effects of opiates on perinatal development have been reviewed elsewhere in the literature (7–13) and in this special issue of *Frontiers in Pediatrics*.

Opiates (derivatives of the opium poppy), such as morphine, which is a major bioactive metabolite of heroin in the brain, can have tremendous therapeutic value for alleviating chronic pain, but can also have significant abuse liability. Opiate drugs act by affecting and interfering with endogenous opioid receptors and peptides (14–20), which are collectively referred to as the endogenous opioid system (20–22). To understand the actions of opiate drugs during development, it is important to appreciate how endogenous opioid neuropeptides and receptors modulate growth and maturation. It is also important to specifically identify how opiate drugs interfere with the endogenous opioid system. Much work remains to be done.

## Opioids are Modulatory

Unlike trophic factors, which can turn cellular processes on or off, e.g., neurotrophins that activate tyrosine receptor kinases (23) or wingless-type mouse mammary tumor virus integration site family (Wnt)/β-catenin signaling (24), that can switch cellular processes on or off, opioids are modulatory, meaning that they adjust biological responses quantitatively rather than qualitatively. This is especially evident during development. While opioids modify rates of ongoing cellular proliferation, differentiation, or death, and can modulate the effects of trophic factors, they do not trigger or discontinue maturational events. Instead, opioids act later in the developmental process, coordinating the timing and numerical matching of neurons and glia within a particular brain region and following the more dramatic actions of trophic factors. In fact, some brain regions that express endogenous opioid peptides and receptors during maturation—do so only transiently and no longer express them in the adult, which supports the notion that opioids are playing a unique role in development. Moreover, endogenous opioid peptides and receptors can be transiently expressed by adult neuronal progenitors in the subgranular zone (SGZ) of the dentate gyrus (discussed below). Thus, the effects of opioids on cellular growth are not restricted to embryonic and early postnatal development but continue to affect cellular maturation throughout ontogeny and are important mediators of adult neuroplasticity.

Another concept for understanding the actions of opioids in maturation is that their actions are largely dependent on context. The effects of opioids can vary depending on the cell-type, duration of exposure or stage of development, and may also depend on pre- or co-exposure to other factors (25). In neurons, for example, morphine-induced μ-opioid receptor (MOR) signaling typically activates extracellular-signal regulated kinase (ERK) 1/2 (26), while prolonged morphine exposure negatively *via* MOR regulates ERK 1/2 signaling in astrocytes (27). Coupling of MOR, δ-opioid receptors (DOR), κ-opioid receptors (KOR), and opioid related nociceptin receptor 1 (also known as the nociceptin or orphanin FQ receptor) to downstream signaling events may be similar or can differ among cell types (28). Despite an abundance of MOR binding early during development, MOR-dependent activation of Gα_i/o_, as assessed by d-Ala^2^-MePhe^4^, Gly-ol^5^-enkephalin (DAMGO)-stimulated [^35^S]guanosine-5′-O-(3-thio)triphosphate ([^35^S]GTPγS) binding, can increase as much as 19-fold from postnatal day 5 compared with some adult brain regions (29). This suggests that MOR receptor-effector coupling may be highly dynamic and vary at different times during maturation (29). In addition to differences in receptor-effector coupling, a highly speculative notion is that the molecular structure of MOR may differ among cell types (30). Multiple MOR polymorphisms and 19 splice variants have been reported (31, 32). MOR-1, MOR-1A, MOR-1X, and MOR-1K splicing variants of the human *OPRM1* gene have been reported to be differentially expressed by neurons, astroglia, microglia, vascular endothelial cells, and pericytes (30).

## Developing Neurons and Glia Can Express Opioid Neuropeptides and Receptors

**Figure 1:**
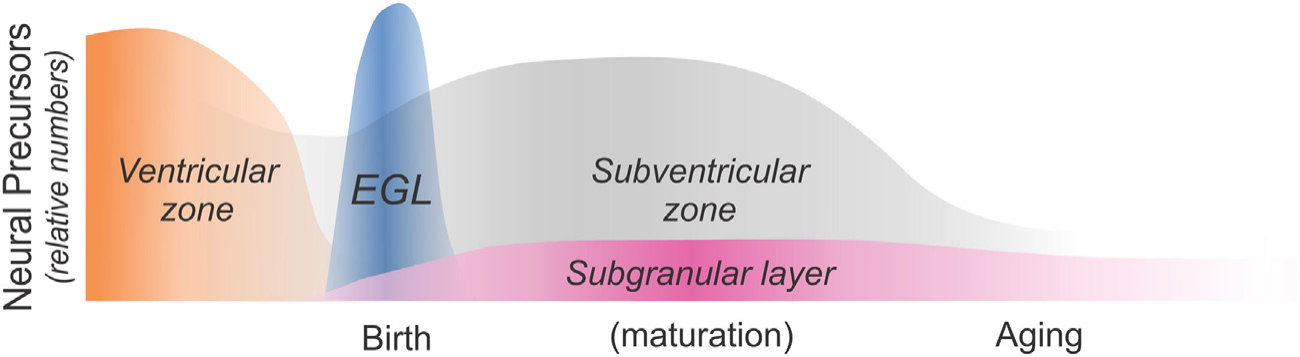
Schematic diagram showing sites of neural precursor production throughout ontogeny. Neural cells are initially produced in the ventricular zone (VZ) and the subventricular zone (SVZ). The cerebellar external granular (or germinal) layer (EGL) is a secondary proliferative zone that arises from the brainstem and exclusively generates neurons (42). The SVZ becomes a major source of macroglia relatively early during maturation (approximately at birth in rodents and during the third trimester in humans), while the subgranular zone (SGZ) of the dentate gyrus is a major site of adult neurogenesis. As discussed in this review article, opiates affect the production and maturation of neurons and/or glia in each of these four regions and at different times throughout ontogeny.

Opioid receptors are expressed by the neural progenitor cells (NPCs) that are the common precursors of all CNS neurons and macroglia, inferring that opioids *per se* might directly influence very early lineage and fate decisions *via* paracrine or autocrine feedback loops. The occurrence of opioid peptides and receptors is not restricted to a particular stage of development, as opioids can be expressed by developing neural cells throughout ontogeny. For example, radioligand binding (33–35), *in situ* hybridization (36, 37), and immunocytochemical (38–40) approaches have all been used to identify MOR, DOR, and/or KOR expression on immature neural cells in the ventricular zone (VZ) and subventricular zone (SVZ) (Figure 1). MOR and KOR transcripts are expressed in murine blastocyst-derived embryonic stem cells (41) and are also present in neural progenitors in SGZ of the adult hippocampus (Figure 1).

Endogenous opioid peptide genes can be transiently expressed during proliferation or differentiation, but not in the mature phenotype, suggesting that the expression is solely related to growth and development. Developing neural cells that temporarily express opioid peptides are particularly intriguing, since transient expression is not associated with the onset of the expression of an adult opioidergic phenotype, but presumably involved in some aspect of cellular maturation, which includes the proliferation, differentiation, and/or programmed cell death of immature neurons and glia or their progenitors. The proteases necessary for cleavage of opioid peptides to bioactive forms, such as those involved in proenkephalin [proprotein or prohormone convertases 1 (PC1) and 2 (PC2) and furin (43)], prodynorphin (*Pdyn*) [PC2 (44)] and processing of proopiomelanocortin (*POMC*)-derived precursors into β-endorphin [PC2 (45–48)], are expressed very early during maturation within germinal zones in the CNS (49). The endogenous opioid peptide gene most frequently expressed by immature neurons and glia is preproenkephalin (*Penk*), whose products can be expressed as partially or fully cleaved peptide products. The expression of proenkephalin peptides can be short-lived and can temporally coincide with cell division in neuroblasts (50–52) and glia (53). The cerebellar external granular layer (EGL) is a transient germinative zone comprised with granule neuron precursors (54, 55). The EGL forms from an outcropping of the SVZ at the rostral portion of the rhombic lips (56, 57). Enkephalin immunoreactivity is transiently expressed by neuroblasts in the EGL (50). EGL neuroblasts express *Penk* mRNA (58, 59), as well as partially processed proenkephalin peptide fragments and the fully processed enkephalin pentapeptide, Met-enkephalin (58, 59). However, the expression of *Penk* mRNA and enkephalin peptides largely disappears as the immature neurons differentiate into adult granule neurons (50, 58), suggesting the expression of *Penk* is unrelated to a mature, terminally differentiated phenotype. This represents a departure from the transcriptional maintenance programs typical of most developing neurons (60) that never switch off and in which continued activity is necessary to maintain the adult transmitter phenotype (61). *Penk* expression by developing astroglia (53, 62–64) and a variety of non-neuronal types during maturation infers that *Penk* expression is important for maturation. Finally, because neural progenitors express opioid receptors it is reasonable to speculate that opioids *per se* might directly influence neural cell maturation *via* paracrine or autocrine feedback. Although there is evidence that applying opioid antagonists alone can enhance growth *in vivo* (discussed below), this is rarely supported by findings in isolated opioid-expressing neural cells *in vitro* where opioid antagonists by themselves only infrequently effect cellular maturation.

Unlike the temporary *Penk* expression patterns that can be present in neuroblasts and immature neurons, *Pdyn* gene products do not appear to be expressed transiently by neuroblasts or immature neurons. Thus, subsets of immature neurons that express *Pdyn* mRNA or Pdyn-derived peptides in the cerebral cortex and striatum (65, 66), hippocampus (66, 67), and hypothalamus (68–70) of rats, retain their dynorphinergic phenotype in the adult. By contrast to neurons, Pdyn-derived peptides are transiently expressed by differentiating immature oligodendrocytes *in vitro* and disappears with maturation (39). *Pdyn* also differs from *Penk* in that *Pdyn* expression is not found in immature or mature astrocytes (53, 62).

The *POMC* gene is less widely expressed in the CNS compared with *Penk* or *Pdyn* genes, and typically in association with areas, such as the hypothalamus (71) and pituitary, that are involved in neuroendocrine function and stress (72, 73). Importantly, however, *POMC* is expressed and post-translationally processed into β-endorphin by adult hippocampal progenitors in the SGZ of the hippocampal dentate gyrus (74, 75) (discussed below).

## Endogenous Opioids Tend to Inhibit Growth

**Figure 2:**
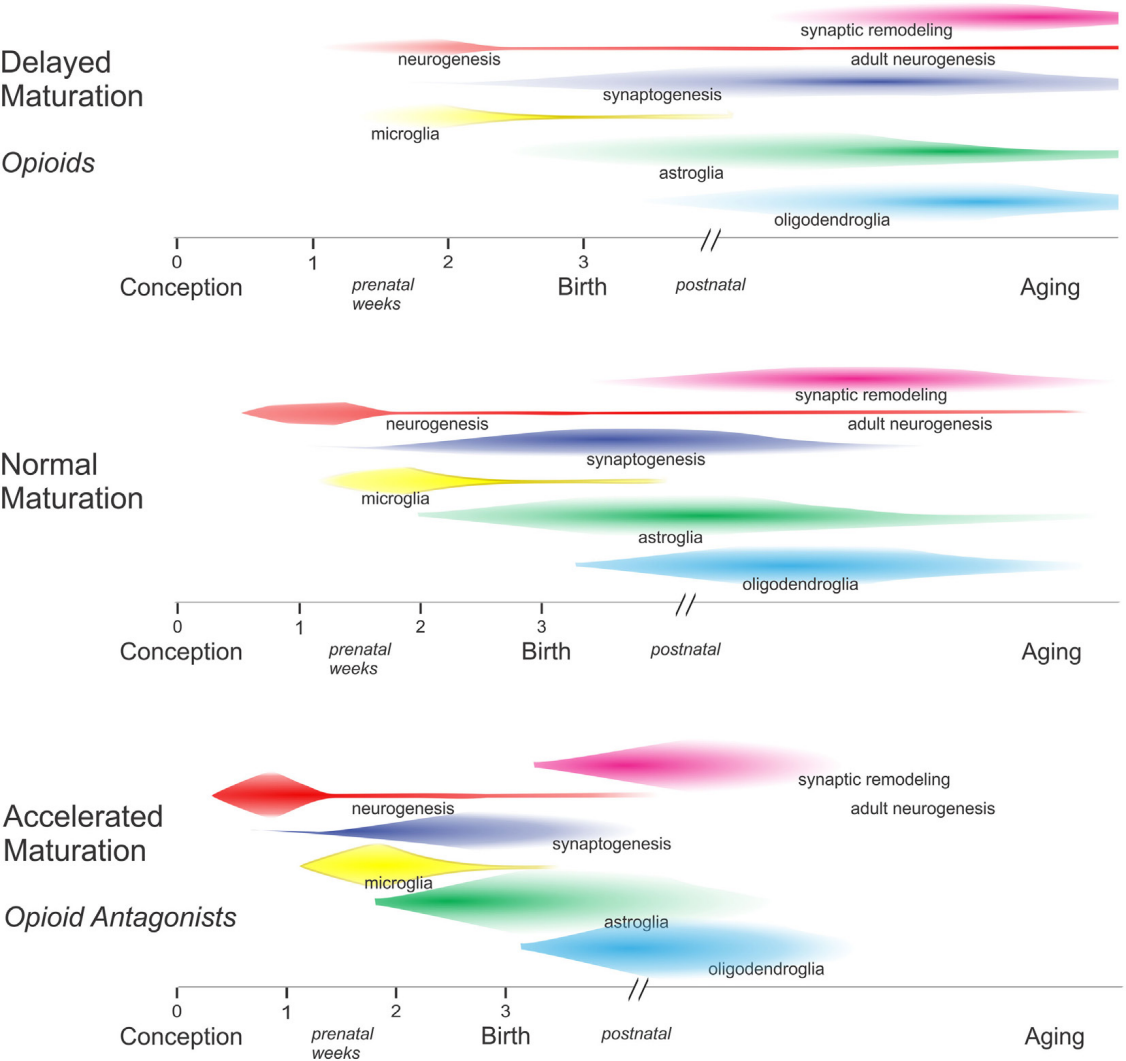
In general, excess opioids can inhibit and delay growth, while opioid receptor blockade can transiently accelerate growth. By inhibiting maturation, opioids decelerate the pace of development among overlapping populations of neurons and glia within specific brain regions. In the absence of opioids, there is some evidence that critical periods may become abbreviated and some developmental events may have insufficient time for completion—resulting in an increased likelihood of errors.

In the context of cellular maturation, endogenous opioids tend to inhibit growth (Figure 2). For example, artificially increasing endogenous opioid levels can reportedly inhibit the proliferation of EGL neuroblasts, while opioid receptor blockade using widely selective opioid receptor antagonists, such as naloxone and naltrexone, increase EGL neuroblast proliferation (39). There are exceptions to the notion that opioids inhibit growth, highlighting the concept that their actions are contextual and cell-specific. For example, acute opioid exposure is mitogenic to immature oligodendrocytes. Both effects on proliferation are discussed in more detail later.

That endogenous opioids inhibit growth is inferred by reports showing that chronic administration of high-dose opioid antagonists to mice and rats can accelerate growth (51, 76, 77) (Figure 2). The long-acting opioid antagonist naltrexone was particularly useful in this regard as a single daily dose of 50 mg/kg in rats or 10 mg/kg in mice was sufficient to continuously block MOR, DOR, and KOR for 24 h (51, 76, 77). Paradoxically, low dosages of naltrexone were found to inhibit growth, presumably because transient exposure to a low dose of naltrexone results in a compensatory upregulation of opioid peptides and receptors (78–80). The experimental evidence for this has not been consistent; the extent to which *Penk* mRNA and enkephalin levels are increased appears to differ among brain regions (78). Importantly, findings from studies with sustained exposure to high-dose opioid antagonists provide circumstantial evidence that the endogenous opioid system is tonically active and can modulate development (51, 77).

Opioid peptides are broadly associated with cellular maturation both within and outside of the CNS, and can affect cellular growth through canonical pathways involving the activation of guanine nucleotide-binding protein (G-protein)-coupled receptors (GPCRs) or by less well-characterized actions such as transcriptional regulation that involve non-canonical mechanisms. Much of our later discussions will be limited to opioid actions at GPCRs, which are the mode of action of abused opiate drugs. However, it is important to appreciate that proenkephalin contains DNA binding domains (81). Proenkephalin transcripts lacking exon 2 have been identified in rat forebrain neurons (82). Experimentally, the exon 2 deletion protein is redirected to the nucleus, presumably due to loss of a normal cytoplasmic-signaling sequence (83). Opioid peptides trafficked in this manner can act as transcription factors (84) to affect cells both within and outside the nervous system. By virtue of its actions as a transcriptional regulator, proenkephalin can regulate the proliferation and direct the fate of T-lymphocytes (84, 85), as well as modulate the production of cytokines such as IL-6 (84). In addition, proenkephalin-derived peptides (86) and the POMC-derived peptide, β-endorphin (87), are expressed by a large variety of cancers. This includes lung cancer cells (88, 89) and neoplastic cells of neural and non-neural origin (90). Opioid receptor activation can suppress the division of cancer cells in culture, while the blockade of opioid receptors can enhance cell proliferation (88, 89) or differentiation, mirroring the observations made in most non-neoplastic cells. Opioid-induced immune suppression, including diminished natural killer cell activity, phagocytosis, and antibody production may also promote tumor growth (91). Few studies have examined the effects of opiate tolerance on cancer cells and whether biological adaptation occurs following sustained exposure. The role of the endogenous opioid system and opiate drugs in neoplasia is inconclusive and has been reviewed elsewhere (92–94).

A key initial step toward understanding the mechanisms by which opioids modulate neuronal and glial maturation is to determine the extent to which opioids act directly or indirectly to alter proliferation, migration, differentiation, or survival of these cells and their progenitors. Since opioids have pronounced effects on a wide variety of physiological systems outside the CNS, such as respiration, gastrointestinal, and endocrine functions, and metabolism, that can in turn affect cell growth and development, determining the direct cellular effects of opioids is critical, and the direct effects of opioid signaling on the major types of CNS cells are considered individually in the following sections.

## Neuroblast Proliferation

**Figure 3:**
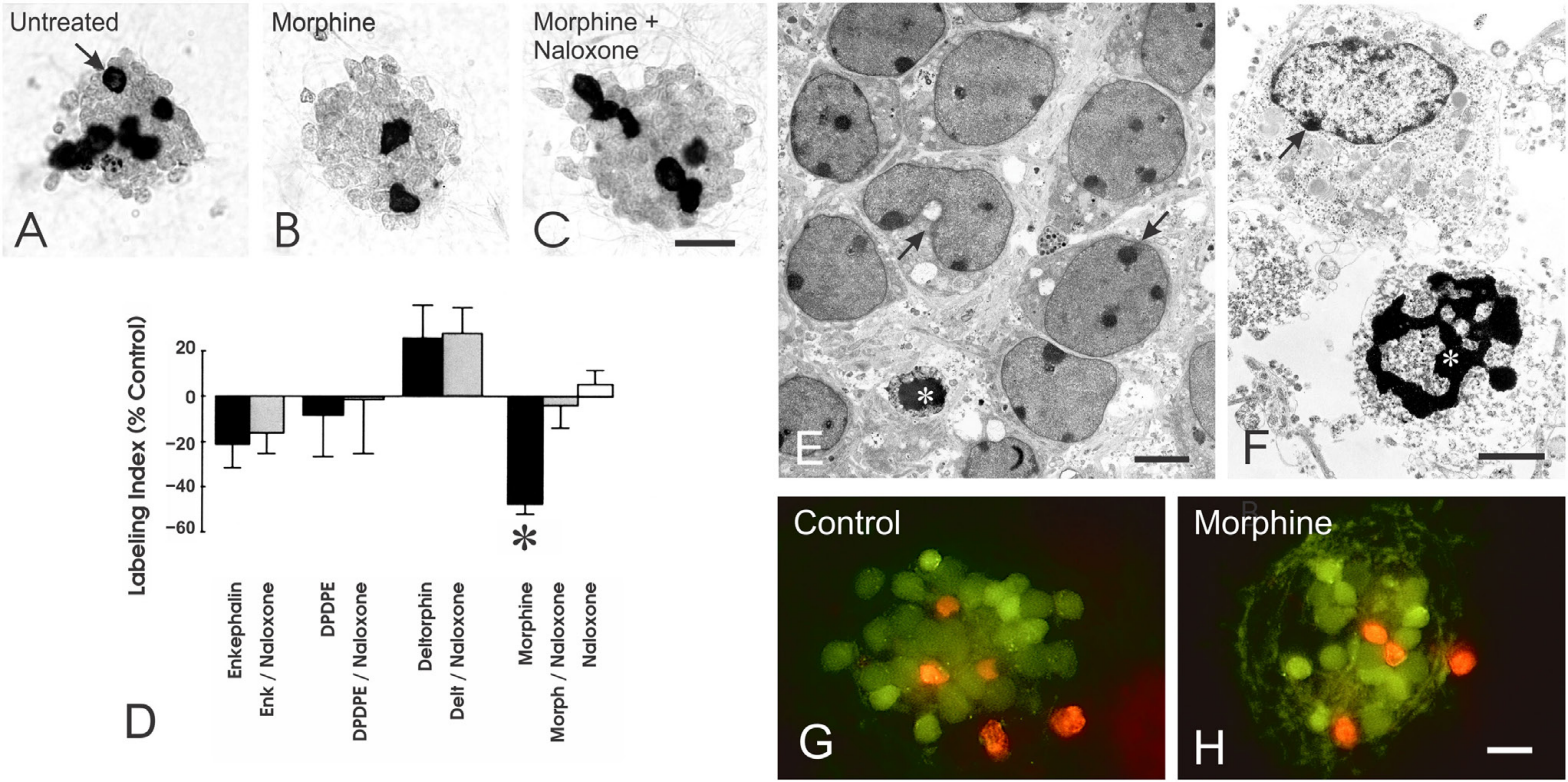
Opioids can directly inhibit the proliferation, but not the survival, of neuronal progenitors in the cerebellar external granular layer (EGL) the cerebellar external granular layer (EGL) *in vitro*. **(A–C)** Neuroblasts from the EGL were isolated using Percoll density gradient centrifugation and reaggregated into neurospheres *in vitro*. Exposure (24 h) to the morphine-induced μ-opioid receptor agonist morphine (Morph), but not the δ-opioid receptor agonists Met-enkephalin (Enk), [D-Pen^2,5^]-enkephalin (DPDPE), or [D-Ala^2^]-deltorphin II (Delt), significantly decreased incorporation of the thymidine analog 5-bromo-2′-deoxyuridine (BrdU) by granule neuron precursors (*p > 0.025 versus untreated controls) **(D)**. Compared with untreated controls **(A)**, continuous morphine treatment (1 µM) for 24 h reduced the proportion of neuroblasts incorporating BrdU [**(B)**; arrow]. The inhibitory effects of morphine were prevented by concurrent administration of naloxone (3 µM) **(C,D)**. Neuroblasts incorporating BrdU were densely labeled (arrow); daughter cells appear near one another after dividing. Data represent the mean ± SEM of *n* = 4–6 experiments. Scale bar = 25 µm **(A–C)**. Effect of opioids on neuronal precursor survival. **(E,F)** Electron micrographs showing large numbers of viable and some degenerating EGL neuronal precursors in untreated cultures. **(E,F)** Compromised neurons displayed cytoplasmic vacuoles and swollen mitochondria [arrows in **(E)**] or accumulated cytoplasmic vacuoles and marginal heterochromatin [arrow in **(F)**], while some dying cells were evident and displayed pyknotic nuclei with shrunken and densely packed heterochromatin [* in **(F)**]. Scale bars = 5 µm **(E)** and 10 µm **(F)**. **(G,H)** Fluorescent images of living (*green fluorescence*) and non-viable (*red fluorescence*) immature neurons from the EGL in untreated control **(G)** and morphine-treated **(H)** neurospheres. Exposure to morphine (1 µM) for 24 h had no effect on EGL cell death (scale bar = 20 µm) **(H)**. Reprinted from Ref. (97); Copyright (2000), with permission from John Wiley and Sons.

To determine whether opioids might be directly affecting the growth of cerebellar EGL neuroblasts, we used a procedure developed by Hatten and coworkers (95, 96) in which enriched populations of EGL neurons are isolated using a two-step Percoll gradient and reaggregated into neurosphere cultures (95, 96). The isolated murine EGL neuroblasts and their granule neuron progeny possessed MOR and DOR, but not KOR, immunoreactivity *in vitro*, as well as proenkephalin immunoreactivity (97). In the EGL cultures, morphine exposure (1 µM) significantly reduced DNA synthesis at 24 h and the number of EGL cells and their granule neuron progeny at 48 h (97) (Figure 3). The inhibitory effects of morphine on DNA synthesis and cell numbers were prevented by naloxone (3 µM) suggesting that the anti-proliferative effects of morphine are due to the activation of specific opioid receptors. By contrast, the DOR agonists Met-enkephalin, [D-Pen^2,5^]-enkephalin (DPDPE) and [D-Ala^2^]-deltorphin II (Delt) (both were added at a 1 µM concentration) had no effect on DNA synthesis at 24 h (Figure 3) or on EGL cell numbers at 48 h (97).

While experiments *in vivo* have not yet fully assessed the direct effects of opioids on individual cell types, many of the *in vitro* findings are at least partly mirrored *in vivo*. Acute opiate exposure typically inhibits the proliferation of neuroblasts (51, 52, 98–105) *in vivo*. Initial studies showed that exposure to morphine or methadone markedly reduced cell numbers in the forebrain during the time when cells are generated from VZ/SVZ (99, 106, 107). Some caution is warranted when interpreting *in vivo* studies that largely rely on measuring the incorporation of thymidine analogs [e.g., [^3^H]thymidine or 5-bromo-2′-deoxyuridine (BrdU)] using biochemical and/or morphologic (counting the number or proportion of [^3^H]thymidine- or BrdU-labeled cells) to assess cell proliferation. Although there is typically a high degree of correlation between increased DNA synthesis and whether a cell subsequently divides, examining DNA synthesis alone can yield an inaccurate picture of cell division (108). Measuring DNA synthesis alone assumes (i) that cells entering the DNA synthesis (S) phase of the cell cycle will subsequently undergo mitosis, (ii) that the duration of the other phases of the cell cycle, or (iii) the incorporation of thymidine itself is not affected by the experimental conditions. Exploring other parameters such as cell death is paramount since neurons in the developing nervous system can be frequently overproduced only to be later culled through programmed cell death (discussed later).

Growth inhibition is not restricted to a particular brain region, stage of development, or opioid receptor type. For example, enkephalinamide reduces [^3^H]thymidine incorporation in the forebrain, hypothalamus, and cerebellum of 11-day-old rats (104). Administering Met-enkephalin (52, 109) or other proenkephalin A derivatives (109) similarly attenuated DNA synthesis brain within the cerebellum and medullary area in 6-day-old rats. In a more detailed study, administering MOR (DAMGO), DOR [(d-Ser^8^)-leucine enkephalin-Thr (DSLET)], or KOR (bremazocine) agonists caused time-dependent (2.5, 4.5, and 8.5 h post-injection) alterations in [^3^H]thymidine labeling and mitotic indices in germinal cells of the cortical VZ of embryonic day 16 rats exposed *in utero* that differed among MOR, DOR, or KOR agonists and that displayed subtle differences between right and left hemispheres (38). In general, the MOR agonist DAMGO was mitogenic at 8.5 h and to a greater extent at 4.5 h post-injection, while DOR activation with DSLET decreased [^3^H]thymidine labeling and mitotic indices at 4.5 h post-injection suggesting the duration of the S and the mitotic (M) phases of the cell cycle are reduced (38). This study demonstrated the widespread and coordinated actions of the endogenous opioid system in modulating the generation of new neurons in the VZ or SVZ. Furthermore, the results provided novel evidence that KOR activation may regulate the development of circuitry involved in the lateralization of the brain (38).

A speculative notion is that the actions of KOR agonists during maturation may partially underlie the neurochemical and functional (right versus left)-asymmetry seen in the enkephalinergic and dynorphinergic systems in the adult CNS of rodents (110–113) and humans (114). For example, the lateralization of enkephalinergic and dynorphinergic processing in the anterior cingulate cortex is hypothesized to be involved in the differential regulation of positive and negative emotions and in the perception of pain in the right- versus left-hand sides of the brain (114). Alterations in Pdyn-derived peptide processing, dynorphin levels, and KOR signaling in forebrain areas including the prefrontal cortex and striatum have been implicated in substance abuse and pro-addictive behaviors (115–118). Enduring changes in these systems during maturation are likely to cause lasting alterations in neural circuitry underlying addiction, as well as other CNS functions unrelated to addiction.

The developmental effects of opioids on the cerebellar EGL have been explored in more detail than in many other regions. Opioid peptides and receptors are highly expressed by the immature EGL cells (consisting of neuroblasts and immature, postmitotic neurons), while the mature granule neurons derived from this germinative zone lose this expression (34, 50, 58). Thus, the opioid system in the EGL is positioned to be more involved in maturational processes and less related to the development/maintenance of adult neurochemical systems (42). Manipulation of the opioid system alters the proliferation of EGL neuroblasts and production of granule neurons derived from the EGL (51, 52, 77, 105). Morphine or methadone, which act preferentially at MOR, reduced thymidine incorporation and/or resulted in reductions in EGL cell numbers *in vivo* (119–121). Alternatively, despite findings that opioid receptor antagonists augment EGL cell proliferation *in vivo* (51) and that EGL cells express proenkephalin peptides *in vitro* (97), neither the broad-spectrum opioid receptor antagonist naloxone nor selective DOR antagonists alone affected the proliferation of isolated EGL cells (97). Findings in isolated EGL cells suggest that opioids do not directly affect EGL neuroblast growth through autocrine and/or paracrine feedback. Instead, opioids may affect another aspect of cerebellar maturation, such as Purkinje cell development, which in turn can influence EGL cell growth (122, 123).

## Neuronal Differentiation

Unlike the effects of morphine on EGL proliferation, morphine had no effect on granule neuron differentiation (97). Alternatively, dendritic growth was stunted by 48 h exposure to the selective DOR agonists enkephalin (1 µM) or Delt (1 µM), but not the selective DOR agonist DPDPE (97). The fact that distinct DOR agonists act differently at the same receptor strongly infers that the effects of DOR agonists on neuronal differentiation are functionally selective and displaying “biased agonism” (124–126), meaning that individual ligands selectively stimulate different signal transduction pathways *via* the same receptor. Finally, the findings also provide clear evidence that different opioid receptor types, MOR or DOR, can independently regulate different aspects of development in the same cell.

**Figure 4:**
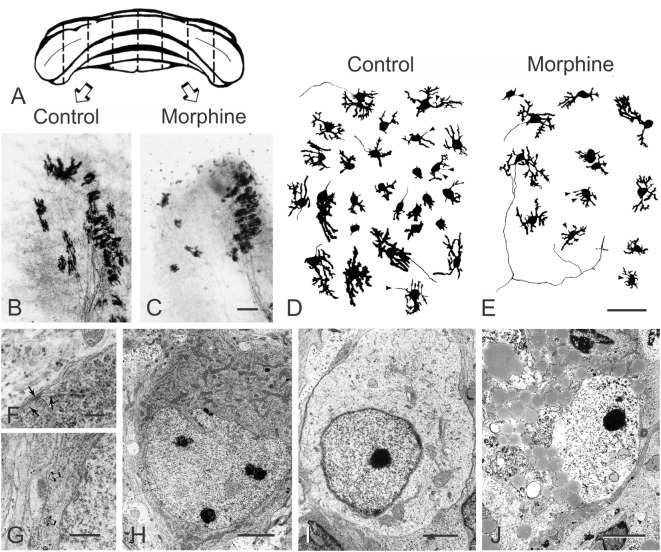
Morphine inhibits Purkinje cell dendritic differentiation and increases their death in a concentration-dependent manner in organotypic cultures of the mouse cerebellum. **(A–C)** Bilaterally matched explant pairs were grown in the presence and absence of morphine. **(A)** Schematic drawing of the cerebellum from a 1-day-old mouse illustrating the “homologous- or mirror-pair” paradigm (129, 130) used to assess the experimental effects of opiates in this study (127). **(B,C)** Bilaterally matched explant pairs are matched in size, shape, and cytoarchitecture and this is maintained by organotypic culture conditions. Purkinje cells in the untreated control explants **(B)** had larger dendrites compared with their matched-pair counterparts continuously treated with morphine (1 µM) **(C)** for 7–10 days [scale bar = 25 µm; **(B,C)** are the same scale]. **(D,E)** Composite camera lucida drawings of calbindin-D_28k_-immunoreactive Purkinje cells illustrating the effects of high concentrations (10 µM) of morphine on Purkinje cell numbers. Compared with untreated controls **(D)**, continuous exposure to morphine **(E)** for 7–10 days *in vitro* caused concentration-dependent reductions in dendritic length and in the number of calbindin-D_28k_-immunoreactive Purkinje cells in cerebellar explants derived from 1-day-old mice. Filopodial processes (arrowheads) extend from the cell body or dendrites of developing calbindin-D_28k_-immunoreactive Purkinje cells—some filopodia terminate in growth cones (arrowheads) [scale bar = 25 µm; **(D,E)** are the same scale]. **(F–J)** Electron micrographs of Purkinje cells in organotypic explants continuously exposed to morphine for 7–10 days *in vitro*. Many Purkinje cells in morphine-treated organotypic explants display normal ultrastructural features. Cerebellar Purkinje cells can be identified by unique features, such as subsurface or hypolemmal cisternae [arrows in **(F)**] (131–133) (scale bar = 1 µm). **(H)** Developing Purkinje cell (scale bar = 5 µm). **(G)** Higher magnification of the axosomatic synapse contacting the Purkinje cell in [**(H)**; arrows] (scale bar = 2 µm). **(I,J)** Degenerating cells in morphine-treated organotypic explants at 7–10 days *in vitro*. **(I)** A degenerating Purkinje cell with a deficit in cytoplasmic organelles and abnormally dense marginal heterochromatin (scale bar = 2 µm). **(J)** A large, dying neuron resembling a Purkinje cell with access lipid inclusions and glycogen in the cytoplasm and partial destruction of the nuclear membrane (scale bar = 3 µm). With progressive degeneration and loss of cellular morphology, it can be difficult to identify Purkinje cells with certainty. Reprinted from Ref. (127); Copyright (1994), with permission from Elsevier.

Unlike the effect of morphine in EGL neuroblasts, morphine can inhibit the differentiation of Purkinje cells in organotypic explants from 1-day-old mice (127). At 7–10 days *in vitro*, calbindin-D_28k_-immunoreactive Purkinje cells in control, as well as morphine-treated explant cultures, possessed dendrites with growth cones and filopodial processes indicative of active growth (Figure 4). The presence of developing synapses was also confirmed by using electron microscopy (Figure 4). Continuous exposure to morphine (Figure 4E) for 7–10 days *in vitro* caused concentration-dependent reductions in the length of growing dendrites in cerebellar explants derived from 1-day-old mice. Exposure to morphine for 7–10 days resulted in a significant reduction in dendritic length (EC_50_ = 49 nM) and the effects of a high (10 µM) concentration of morphine were completely reversed by concurrent naloxone administration. Importantly, the effects were shown to be concentration-dependent and preventable by naloxone suggesting that the effects of morphine are mediated by specific opioid receptors. Although the cellular site(s) of morphine’s actions within the cerebellum are uncertain, the results clearly indicate that morphine, through direct actions on the cerebellum *per se*, can inhibit the differentiation of Purkinje cell dendrites. Morphine can directly reduce EGL cell proliferation and granule cell numbers (97), and given the well-known trophic ability of granule cells to direct Purkinje cell maturation (128) and known interdependence between these two cell types (122, 123), this is strong evidence that morphine modulates granule cell and Purkinje cell trophic interactions during development.

The cerebellum is becoming increasingly linked to neural circuitry involved in addictive behaviors including aversion, reward, motivational drive, and saliency (134, 135). Addiction is associated with “impaired response inhibition” and “salience attribution” (136–138). Emerging evidence suggests that the cerebellum maintains the homeostatic balance among these brain regions (134, 135), and is critical for habit formation, attention to novel stimuli, and behavioral inhibition—behavioral traits associated with addiction (135). Moreover, chronic exposure to a variety of substances in adults can result in structural deficits in the gray matter and/or white matter within cerebellar lobules VI, VIIb, Crus I, Crus II, and within the vermis (134, 139). Assuming greater sensitivity of developing versus the mature cerebellum, perinatal exposure to opiates is likely to have profound organizational effects on the neural circuits regulating drug taking behavior. Importantly, not only do Purkinje cells in the cerebellar vermis transiently express *Penk* mRNA and enkephalin immunoreactivity during development (58, 59), but also the dendritic complexity and/or the density of spines of Purkinje cells within the vermis of cerebellar lobule VIII in 10-day-old rats and lobules VI–VIII in 21-day-old rats is altered in response to prolonged opioid antagonist exposure during maturation (21). The extent to which the transient developmental increases in opioid expression in cerebellar neurons influence adult CNS functions is uncertain.

Exposure to morphine (100) or methadone (140) during development inhibits the neurochemical (140) and morphological (107, 120) maturation of the brain, including reductions in neuronal numbers (106). Hammer and coworkers demonstrated that sustained morphine (10 mg/kg/day) exposure *via* subcutaneous osmotic minipump from gestational day 12 until postnatal day 6 in rats results in significant reductions in neuronal density and in the absolute numbers of neurons in layers II–III, IV, and V of the primary somatosensory cortex, but not layer VI (103). The same study found no effect of morphine on the thickness of layers II through VI of the somatosensory cortex. Interestingly, sustained exposure to the opioid antagonist naltrexone (10 mg/kg/day) also decreased neuronal density in cortical layers II through V and the total number of neurons in layer V, while increasing the thickness of layers II–III and throughout the entire cortex (103). Possible explanations for similar actions of morphine and naltrexone might include: (i) Naltrexone may act by transiently enhancing dendritic differentiation in rat somatosensory cortex (21) and/or (ii) by increasing glial numbers (39). Either action would result in a decrease in the relative density and number of neurons per unit volume, while increasing the overall thickness of the cortex. Earlier work by Hammer and coworkers (141) established that exposure to morphine during perinatal development decreased the length of basilar dendrites of layers II–III pyramidal neurons in somatosensory cortex. Morphine-dependent reductions in basilar dendritic complexity were prevented by co-administering naltrexone (141). In confirmatory studies, the offspring of pregnant rats exposed to morphine (5 mg/kg first 3 days; 10 mg/kg thereafter) during gestational days 11–18, showed enduring reductions in dendritic complexity in layer II/III pyramidal neurons of the secondary visual cortex at postnatal day 25 (142). Decreases in pyramidal cell numbers in hippocampal areas CA1, CA2, and CA3 are also reported in postnatal day 18 or day 32 offspring of female mice chronically administered morphine (10 mg/kg/day) before mating, during gestation, and postnatally (143). More recently, postnatal morphine exposure (2 mg/kg, b.i.d.) from postnatal days 3 through 7 in rats was found to decrease incorporation of the thymidine analog BrdU by NPCs in the dentate gyrus of the hippocampus (144). In addition, the same study detected lower levels of glutamic acid decarboxylase (GAD), taurine, and *myo*-inositol by functional nuclear magnetic resonance spectroscopy, and decreased amounts of myelin basic protein (144) (important for myelinogenesis) in the hippocampus (144). Reductions in GAD, essential for the interconversion of glutamate to γ-aminobutyric acid, suggest marked imbalances in excitatory versus inhibitory neurotransmission.

The accelerated maturation seen with opioid receptor blockade can seem dramatic during peak periods of development in the cerebral cortex, hippocampus, and cerebellum. However, the effects are often transient and in many brain regions untreated controls appear to catch-up during later development (21).

## Neuronal Cell Death

The effects of opioids on cell death and survival in neural and non-neural cells, especially in relation to immune and endocrine function, chronic pain, and cancer in adults have been studied (145–147) and extensively reviewed previously (148).

Purkinje cell death is evident in organotypic explants from 1-day-old mice exposed to pharmacological concentrations of opiates (127). Continuous exposure to high concentrations of morphine (>1 μM) for 7–10 days *in vitro* caused reductions in the number of calbindin-D_28k_-immunoreactive Purkinje cells in cerebellar explants derived from 1-day-old mice (127). The ability of morphine to decrease the number of calbindin-D_28k_-immunopositive Purkinje cells was concentration-dependent (EC_50_ = 3.6 µM) and morphine-induced (10 µM) death in Purkinje neurons was antagonized by co-exposure to naloxone. To assess whether reductions in Purkinje cell numbers might potentially be due to a morphine-dependent decline in calbindin-D_28k_ expression, electron microscopy was used to confirm that Purkinje cell death was occurring especially in morphine-treated explant cultures (Figure 4). Although morphine was cytotoxic to Purkinje cells in organotypic explants from 1-day-old mouse cerebella, toxicity was not evident in Purkinje cells in explants taken from 7-day mouse cerebella (127). This suggests that, even at high concentrations, morphine is not inherently toxic to Purkinje cells; rather, morphine is likely to be interfering with critical developmental events affecting survival during the first, but not second, week of postnatal development in mice. Although cell death is not seen with concentrations of morphine exceeding 1 µM in cultured mouse EGL cells (97) (Figure 3), prolonged (5-day) exposure to a range of morphine concentrations between 10^−12^ and 10^−4^ M induces classic apoptosis, including DNA fragmentation, in human fetal brain neurons (147). This suggests that the duration of exposure to opiates may also be important in triggering programmed cell death (147). The varied response among neuron types and conditions highlights that the mechanism(s) and pathways underlying opiate-induced cell death are far from being completely understood, and likely vary among cell types and context. Other investigators have demonstrated that opiates can modulate cell death *via* PI3K and ERK 1/2 signaling in Chinese hamster ovary or HEK293 cells stably transfected with the murine MOR (149). In some instances, opiates may affect cell survival in addition to their effects on proliferation, although this is not a consistent observation. Alternatively, surrounding glia, which can display extreme regional and developmentally diverse phenotypic patterns of MOR, DOR, and KOR expression (150, 151) (discussed below), may also contribute to varied developmental responses among different neuronal types to opioids.

The death of neuronal precursors is occasionally reported as a consequence of exposure to opiates (152) or endogenous opioids (153) by themselves. In addition, opioids can modulate the effects of existing apoptotic signals [especially in immune cells (154, 155)], and opiates may be cofactors in regulating neural cell death (156, 157) [see Ref. (148)]. Cell death is evident in cultured Purkinje cells with more chronic 7-day exposure to morphine (discussed above) (127), and Purkinje cell losses have been reported in chronic heroin abusers who are HIV-seronegative (158).

## Glial Maturation

The generation of neurons throughout the CNS is thought to be essentially complete at birth in rodents apart from the formation of interneurons of the olfactory bulbs, the dentate gyrus, and the cerebellar cortex (159–162). Nevertheless, in the 1980s, there were emerging reports demonstrating opioid-dependent alterations in DNA synthesis and cell numbers, without changes in cell survival, in neural cells throughout the brain long after peak periods of neurogenesis (98, 100, 104). In fact, in adult rats, increases in [^3^H]thymidine labeling in the SVZ were evident 1 h after morphine injection (10 mg/kg, s.c.) (163) in brain regions that are not associated with adult neurogenesis. This discrepancy prompted us to question whether opioids might have widespread effects on the generation of glia throughout the CNS.

**Figure 5:**
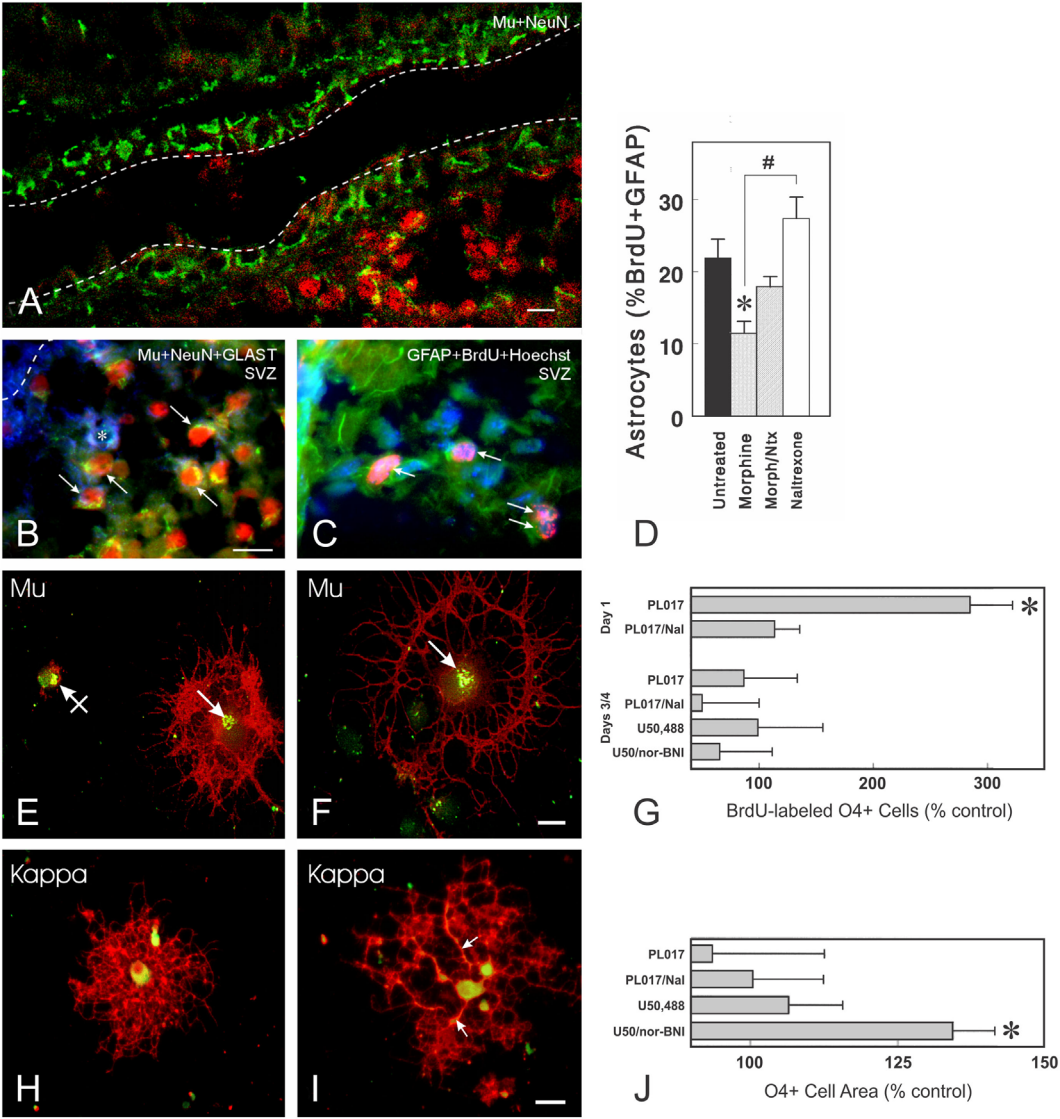
Opioid receptors can be expressed by immature neurons, astrocytes, and oligodendrocytes. **(A)** μ-Opioid receptor (MOR) (Mu; *green fluorescence*) immunoreactive cells can be present in some portions of the ventricular zone and/or subventricular zone (SVZ) of postnatal day 5 mice (scale bar = 20 µm). Many of the MOR+ cells entering the SVZ are NeuN-immunoreactive neurons (*red fluorescence*). The dashed lines represent the borders of the lateral ventricle. **(B)** Photomicrograph showing combined neuronal nuclear (NeuN; *red fluorescence*), glutamate transporter (GLAST, also known as excitatory amino acid transporter 1; *blue fluorescence*), and MOR (*green fluorescence*) immunofluorescence in cells from postnatal day 5 mice. Subpopulations of both neurons (arrows) and astroglia (*) express MOR. **(C)** Thymidine analog, 5-bromo-2′-deoxyuridine (BrdU) labeled (*red fluorescence*), and glial fibrillary acidic protein (GFAP+) astrocytes (*green fluorescence*) are indicated by the arrows **(C)**; cell nuclei are counterstained with Hoechst 33342 (*blue fluorescence*) [scale bar in **(B)** = 25 µm; **(B,C)** *are the same magnification*]. **(D)** Effect of morphine and/or naltrexone on BrdU incorporation in GFAP-expressing cells within the postnatal day 5-mouse brain. Morphine significantly decreased the proportion of BrdU+ astrocytes (*p < 0.05) and there is a significant difference between morphine-treated and naltrexone-treated mice (^#^*p* < 0.05). **(E–J)** Oligodendrocytes express MOR and κ-opioid receptors (KOR) in a developmentally regulated manner *in vitro*. Oligodendrocytes are labeled using the specific anti-O4 antibody (*red fluorescence*) and colocalized with MOR **(E,F)** or KOR **(H,I)** immunoreactivity. **(E,F)** MOR are localized within discrete perinuclear regions of the cell body (arrows) (*green fluorescence*); areas where O4 and MOR or KOR are colocalized display *yellow fluorescence*. MORs can be associated with oligodendrocytes at different stages of development, including very immature oligodendrocytes with negligible O4 immunoreactivity [crossed arrow; **(E)**] and more mature oligodendrocytes with large process networks [arrow; **(F)**] [scale bar in **(F)** = 10 µm; **(E,F)** *are the same magnification*]. **(G)** The effect of opioid receptor agonists and antagonists on BrdU uptake in cultured O4+ oligodendrocytes of two different ages. At 1 day after enrichment, oligodendrocytes are still relatively immature and express only μ-receptors. A 27-h exposure to the MOR agonist PL017 increases BrdU uptake by nearly 300% compared with control values. In the presence of naloxone (PL017/Nal), this effect is not seen. At 3–4 days after enrichment, neither MOR nor a KOR agonist (U50,488) or antagonist [nor-binaltorphimine (nor-BNI)] affect BrdU uptake in a significant manner. Cells were counted on duplicate coverslips from 4 to 5 different cultures (*p < 0.01; ANOVA with *post hoc* Newman-Keuls testing). **(H,I)** By contrast to MOR, KOR are distributed through the entire cell body cytoplasm. Occasional oligodendrocytes also have KOR-immunoreactivity within regions along cell processes [arrows; **(I)**] [scale bar in **(I)** = 20 µm; **(H,I)** *are the same magnification*]. **(J)** Effects of opioid receptor agonists and antagonists on oligodendrocyte cell area. After 48 h of exposure, the only significant effect is seen with the KOR antagonist, nor-BNI, which increased the average cell area compared with control cells (*p < 0.025) **(J)**. This effect was not seen at 27 h (164). The finding that nor-BNI increases the elaboration of cytoplasmic processes provides circumstantial evidence that endogenous opioids are expressed and inhibit oligodendrocyte differentiation (164). Subsequent studies showed that differentiating oligodendrocytes produce dynorphins and these endogenous KOR peptide ligands appear to preferentially decrease the survival of oligodendrocytes during later maturation (165). Thus, an alternative explanation is that the loss of more mature oligodendrocytes results in a net increase in immature oligodendrocytes and the perception that dynorphin is restricting differentiation. Figures **(A–D)** are reprinted from Ref. (39); Copyright (2001), with permission from John Wiley and Sons. Figures **(E–J)** are reprinted with slight modifications from Ref. (164); Copyright (1998), with permission from John Wiley and Sons.

There were several indications that glial-restricted precursors (GRPs), as well as their astroglial and oligodendroglial progeny, could be a direct target of opioids during maturation. (i) As mentioned, opioid exposure has widespread effects on cell numbers during periods of peak gliogenesis, when most macroglial types in the forebrain are being generated from the SVZ and after the production of neurons is largely complete. The sustained mitogenic effects seen in the postnatal forebrain 8–10 weeks following exposure to naltrexone (98) could hardly be explained by alterations in neurogenesis—since the production of neurons from the SGZ and rostral migratory stream is relatively small compared with the observed alterations in cell numbers. In addition to timing, (ii) opioids affect sites of glial production in the SVZ. (iii) Opioid receptors are often expressed by GRPs and immature astroglia, including radial glia (40), and oligodendroglia, but less frequently by their adult counterparts (39) (Figure 5). (iv) Finally as discussed in detail below, opioids can directly affect the proliferation and differentiation of immature astrocytes and oligodendrocytes isolated *in vitro*. Thus, many of the effects of opioids on cell numbers in the postnatal CNS are largely attributable to enduring alterations in gliogenesis rather than neurogenesis.

## Astrocyte Production and Development

We and others have proposed that astroglia are direct targets for opioid drugs with abuse liability (25, 166–170). Astroglial growth has been shown to be inhibited by opioids *in vitro* and *in vivo* (25, 39, 109, 169, 171–174). Astroglia can express MOR, DOR, and/or KOR (39, 150, 168, 169, 171, 174–178). Opioid receptor expression by astrocytes in primary culture is developmentally regulated, differs among brain regions (150, 173, 175, 177, 179–181), and in the case of DOR, appears to be cell cycle-dependent (150, 179). Activation of MOR, KOR, or DOR causes reduced astroglial proliferation (169, 174, 177). In addition to inhibiting astroglial division, morphine also causes astrocytes to hypertrophy. MOR-dependent inhibition of astroglial proliferation and hypertrophy are mediated by increases in intracellular Ca^2+^ concentration ([Ca^2+^]_i_) (174, 177). In these studies, MOR-dependent increases in [Ca^2+^]_i_ could be elicited by morphine (100 nM) or the more selective MOR agonist H-Tyr-Pro-Phe (N-Me)-D-Pro-NH_2_ (PL017) (10 nM, 100 nM, or 1 µM), and resulted from both extracellular Ca^2+^ influx *via* L-type Ca^2+^ channels and from Ca^2+^ mobilization from intracellular stores (174). The effects of morphine on astroglial proliferation and cellular hypertrophy can be mimicked by artificially increasing [Ca^2+^]_i_ (in the absence of morphine) or prevented by chelating intracellular Ca^2+^. Nifedipine (1 µM) was used to assess whether influx through L-type Ca^2+^ channels might be operative. While blocking influx through L-type Ca^2+^ channels attenuated MOR-dependent [Ca^2+^]_i_ increases in astrocytes, this strategy had no effect on morphine’s ability to inhibit proliferation or stimulate hypertrophy. Thapsigargin and dantrolene were used to assess the role of Ca^2+^ mobilization from intracellular sources. Sustained (24 h) exposure to thapsigargin (100 nM), which blocks sarco/endoplasmic reticulum (ER) Ca^2+^ ATPase (182) and prevents Ca^2+^ sequestration into ER thereby depleting the ER of Ca^2+^, was intrinsically toxic to astrocytes confounding any interpretation of the results. Alternatively, blocking Ca^2+^-induced Ca^2+^ release from intracellular stores using dantrolene (10 µM) selectively prevented morphine-induced decreases in astroglial proliferation and cellular hypertrophy (174). Dantrolene selectively blocks ryanodine receptors (RyR) by blocking Ca^2+^ channels associated with the RyR1 and RyR3, but not the RyR2, isoform (183). The results indicate that opiates regulate astroglial growth and maturation through a pathway involving MOR-dependent mobilization of [Ca^2+^]_i_ *via* RyR1 and/or RyR3.

In flat-polyhedral (type I) astroglia isolated from the cerebral forebrain of postnatal day 1–4 ICR mice, KOR-mediated increases in [Ca^2+^]_i_ were initiated using the selective agonists *trans*-(+/−)-3,4-dichloro-*N*-methyl-*N*-[2-(1-pyrrolidinyl)cyclohexyl]benzeneacetamide hydrochloride (U50,488H) (10 nM, 100 nM, or 1 µM) or (+)-(5α,7α,8β)-*N*-methyl-*N*-[7-(1-pyrrolidinyl)-1-oxaspiro[4.5]dec-8-yl]-benzeneacetamide (U69,593) (10 nM, 100 nM, or 1 µM) (184, 185). The effects of U69,593 on astroglial proliferation and hypertrophy could be mimicked by artificially increasing [Ca^2+^]_i_ in the absence of drug and could be prevented by the KOR antagonist nor-binaltorphimine (nor-BNI). Process-bearing (type II) astrocytes, which resemble radial glia, isolated from postnatal day 1–3 mouse spinal cord, widely expressed KOR. Unlike the results in type I astrocytes from the cerebral forebrain, exposing type II spinal cord astrocytes to the KOR agonist U50,488 (1 µM) for 3 DIV or 6 DIV increased DNA synthesis as assessed by BrdU incorporation *in vitro*, while concurrent administration of the KOR antagonist nor-BNI or the use of KOR^−/−^ astrocytes negated the effects of U50,488 (186). A similar mitogenic effect was noted in serum-starved (28 h) astrocytes isolated from cerebral cortex of postnatal day 1 Sprague–Dawley rat pups or from immortalized rat cortical astrocytes (CTX TNA2; ATCC) exposed for 24 h to U69,593 (1 µM) or the KOR agonist derived from salvinorin A, MOM-Sal-B (1 µM) (187). The collective findings suggest that KOR activation can have both inhibitory and excitatory effects on astroglial proliferation and that this may differ among astroglial types, brain regions, and context. Inhibition of astrogliogenesis through MOR-and KOR-mediated events was shown to be driven *via* ERK, by contrast to the implication of the p38 mitogen-activated protein kinase pathway in opioid-mediated attenuation of neurogenesis (41). Importantly, opioids can modulate basic fibroblast growth factor and/or epidermal growth factor activation of ERK in C6 glioma cells or in primary astrocytes (170, 188, 189). Whether DOR-dependent increases in astroglial [Ca^2+^]_i_ are also antimitotic have not been explored. The “reactive” cellular hypertrophy is accompanied by increased glial fibrillary acidic protein (GFAP) immunoreactivity and aspects may mimic reactive gliosis *in vivo* (174, 190).

In 4–5-day-old postnatal mice, 4.5 h of morphine (20 mg/kg s.c.) exposure significantly decreased BrdU incorporation by astrocytes in the SVZ of the lateral ventricle (39). A similar slowing of proliferation was suggested in a study where the G_2_/M phase of cell cycle was lengthened in GFAP+ radial glia in the cerebral cortex when gestational day 15.5 (E 15.5) embryonic mice were exposed to morphine (E 15 pregnant dams received 3 × 10 mg/kg s.c. at 3 h intervals) (191). Based on findings that opioid receptor activation can regulate cellular development, it seems probable that the varied patterns of opioid receptor and peptide expression within diverse cell types permit the simultaneous and coordinated control of development within heterologous subpopulations of cells. Moreover, findings that many NPCs likely express MOR, and that morphine acutely alters astroglial development, prompt speculation that chronic opiate exposure might cause lasting changes in neural function by disrupting gliogenesis.

Morphine normally has little or no effect on astroglial viability during maturation after either acute or sustained exposure (192). Indeed, morphine can protect neonatal astrocytes against the cytotoxic effects of peroxynitrite (193). Similarly, astroglial apoptosis, as assessed by caspase-3 cleavage, was not evident in neonatal rat pups exposed to morphine (10 mg/kg; b.i.d.) on postnatal day 1 through 7 (194). Alternatively, morphine-induced death is evident in astrocytes in mixed-NPC cultures from 14-day mouse embryos (195). Although astrocyte death is not reported with exposure to opiates alone *in vitro*, sustained (96 h) exposure to morphine (500 nM) causes small, but nevertheless significant, increases in the death of immature astrocytes or glially restricted precursors that have been co-exposed to the HIV-1 Tat (transactivator of transcription) protein (100 nM) (156). This and other evidence suggests that the response of target cells to morphine can be “reprogrammed” by exposure to HIV-1 Tat. Morphine (and presumably other opiates) can act as biological response modifiers, imparting very different intracellular signals that instruct some astrocytes to die if they have been pre- or co-exposed to HIV-1 Tat (156), and perhaps other biological stressors. These findings illustrate the importance of context in shaping how developing neural cells interpret an opioid signal.

## Oligodendrocytes

Oligodendrocytes produce myelin in the CNS (196) and originate during a second wave of glial maturation following genesis of astroglia (197, 198). Slow generation of new oligodendrocytes and adaptive myelination are processes that continue throughout life (199). Importantly, chronic opiate exposure results in significant, selective disruption of white matter tracts throughout the CNS (200–203). While some of this injury may be due to indirect effects involving inflammatory changes or loss of support from astroglia, it is also clear that significant effects of opiates occur directly on oligodendroglia. Our labs were the first to show that oligodendrocytes express MOR and KOR (164, 204). Unlike the situation in neuroblasts and astroglia, where MOR activation inhibits cell replication, MOR activation appears to be mitogenic in immature oligodendroglia since proliferation as assessed by BrdU was stimulated by the MOR-selective agonist PL017 (1 μM) and prevented by co-administration of naloxone (3 μM) (204). By contrast to MOR, KOR begins to be expressed in more mature, non-dividing oligodendroglia (164). KOR blockade with nor-BNI, in the absence of exogenously added opiates, increased the size of myelin-like membranes in cultured oligodendroglia (164), suggesting that the cells themselves might produce KOR agonists. This was later borne out by studies showing production and release of *Penk*- and *Pdyn*-derived peptide products by cultured oligodendroglia (165). Nor-BNI greatly enhanced glutamate-induced death of oligodendroglia while causing only modest effects on its own, again invoking the issue of context in opiate effects on survival as well as growth/differentiation (165). A relationship between KOR and oligodendrocyte function/myelin production was also suggested by our finding that KOR (but not MOR) was severely reduced on oligodendrocytes, but not neurons, in the jimpy mouse, a dysmyelinating mutant (205). More recent work has also shown that KOR agonists, including U50,488, can promote differentiation/myelination in human oligodendrocytes derived from immortalized pluripotent stem cells (206). Thus, similar to granule neurons, different aspects of oligodendroglial development appear to be independently regulated by opioid signaling. However, unlike granule neurons and their EGL precursors, which do not appear to express KOR *in vitro* (97), MOR activation in immature oligodendroglia is mitogenic (164).

More recently, chronic, 9 days MOR or KOR activation was shown to increase the production of young oligodendrocytes during the sequential differentiation of murine embryonic stem cells, seemingly at the expense of neurogenesis and astrogliogenesis (207). Effects of the MOR-selective agonist DAMGO (1 µM) or the KOR selective agonist U69,593 (1 µM) were similarly dependent on ERK and p38 mitogen-activated protein kinase (MAPK) signaling pathways.

Buprenorphine is a newer treatment for opiate addiction than methadone that was initially thought to have fewer side effects, less abuse liability, and greater safety because of its actions as a partial MOR agonist (208–211). In addition to its actions at MOR, buprenorphine can act as a partial antagonist at KOR (212) and as an agonist at nociceptin/orphanin FQ (NOP or OLR1) receptors (212, 213). Though beneficial in treating pregnant addicts (214–218), like methadone, buprenorphine can cross the placenta (219), has abuse liability (220–222), and at high doses it can have variable effects on neurogenesis (223, 224). Prenatal exposure to large dosages of buprenorphine can reduce myelin basic protein levels and the number of myelinated axons (213) and can trigger depressive-like behavior (225) in rats. The aberrant myelin patterns seen with high concentrations of buprenorphine are attributed to its actions as a partial KOR antagonist, although possible agonist actions at ORL1 receptors, which have an emerging role in CNS plasticity (226), cannot be overlooked.

Methadone is also beneficial as an addiction therapy during pregnancy. Unlike buprenorphine, methadone is a preferential MOR agonist with far fewer actions at KOR. Methadone crosses the placenta (227) and some methadone is present in milk during lactation (228). Recent evidence from the investigators that studied the effects of buprenorphine on white matter development described above, indicates that methadone also affects oligodendrocyte maturation and myelination. Gestational day 7 pregnant rats were continuously administered subcutaneous methadone (9 mg/kg/day) *via* osmotic minipumps throughout the pregnancy and during lactation until the day of sacrifice on postnatal days 11 or 19. This early methadone exposure significantly increased oligodendrocyte proliferation and differentiation, including the production of myelin basic protein, myelin proteolipid protein, and myelin-oligodendrocyte glycoprotein (229). Myelin ultrastructure was markedly affected. Methadone exposure (24 h) caused concentration-dependent increases in DNA synthesis in oligodendrocyte progenitors in cell culture (229), similar to what we had previously observed with the selective MOR agonist PL017 in immature oligodendrocytes *in vitro* (164, 204). Finally, these results suggest that increases in [^3^H]thymidine labeling in the SVZ 1 h after morphine injection (10 mg/kg, s.c.) in adult rats mentioned earlier (163) may be due to a mitogenic effect of morphine on adult oligodendrocyte precursors, since immature astroglia show reduced proliferation following acute morphine exposure (39). The collective findings indicate that, unlike the response of neuroblasts and immature astroglia in which acute opiate exposure inhibits proliferation, MOR receptor activation can be mitogenic to young oligodendrocytes and their progenitors.

## Inhibition of Adult Neurogenesis and CNS Plasticity

Although the intention of this review is to emphasize the role of opiates during development, opiate-dependent reductions in synaptodendritic complexity are not restricted to the pre- and post-natal brain maturation periods. The adult CNS remains highly plastic and modifiable with sustained opiate exposure; however, unlike the situation during development, the effects of opiates on dendritic culling in the mature brain tend to be less severe. Since several authoritative reviews on the effects of opioids (230–234), as well as other substances with abuse liability (235), on adult neurogenesis have been published, we will only minimally address the topic here.

Adult neurogenesis was first described in rodents (236, 237) and later described in nonhuman primates (238) and humans (239). In mammals, adult neurogenesis is thought to be restricted to two specialized “neurogenic” regions within the SGZ of the hippocampal dentate gyrus and within the SVZ of the lateral ventricle. Adult neurogenesis in both regions (the SGZ and the SVZ) declines with aging (240). Newly generated neurons within the SGZ become granule neurons within the dentate gyrus, while neurons produced within the SVZ of the lateral ventricle migrate through the rostral migratory stream to become interneurons in the olfactory bulb in rodents and nonhuman primates (241–243). In humans, new SVZ neurons do not appear to migrate into the olfactory bulb, but instead remain as interneurons within the striatum (244). Adult hippocampal progenitors derived from the rat SGZ express the endogenous MOR peptide β-endorphin—a post-translational product of the *POMC* gene (245). In fact, POMC expression has been used as a marker for adult neural progenitors in the hippocampus (246). Adult hippocampal neural progenitors can be visualized in mice in which an enhanced-green fluorescent protein reporter is driven by *POMC* expression (246).

To what extent do opiate drugs directly affect the maturation and fate of adult neural progenitors? In the dentate gyrus, adult neurogenesis is sustained by a complex relationship between environmental factors imparted by adjacent neural cell types within the unique neurogenic niche of the SGZ (247–250). Although it may not be possible to preserve the complex spatial relationships [especially between the vasculature and the neurogenic niche (251, 252)] necessary to maintain the unique milieu of the neurogenic niche in cell culture (243, 251–253), acutely dissociated (in which physical cell-to-cell interrelationships are lost), reaggregate neurospheres (254), or organotypic (in which the vascular niche is no longer functional) cultures of adult SGZ neural progenitors continue to divide and retain key phenotypic characteristics permitting some experimentation *in vitro*.

The effects of morphine on adult hippocampal neurogenesis were first described by Eisch et al. (255). In these studies, adult rats received chronic morphine either subcutaneously (75 mg morphine time-release pelleted implant) for 5 days or *via* self-administration for 26 days. Importantly, morphine received *via* either delivery route had sustained reductions in BrdU-labeled neural progenitors in the dentate gyrus, granule cell layer, and/or hilus of the hippocampus, thus indicating reductions in adult neurogenesis (255). Among other outcomes, this effect may have consequences for drug-seeking behavior, as illustrated by the finding that radiation-induced destruction of adult neural progenitors increases morphine, but not sucrose, self-administration (256). This interesting result suggests that chronic opiate abuse, by limiting the pool of adult neural progenitors, may restrict the formation of new neural circuitry that is beneficial in limiting drug-seeking behavior (256).

Morphine-dependent decreases in neurogenesis have been more specifically attributed to increased numbers of neuroblasts being retained in the S phase of the cell cycle, as well as fewer young postmitotic neurons exiting the cell cycle (257). It was also suggested, based on cleaved caspase-3 immunoreactivity, that increased numbers of proliferating neuroblasts were dying (257); however, immunocytochemical evidence of caspase-3 cleavage is not definitive evidence of impending cell death since caspase-3 can be partially activated to intermediate levels during cellular remodeling without triggering downstream effectors, such as caspase activated DNase, necessary for cell death (258).

β-endorphin influences the growth of SGZ progenitors through autocrine and/or paracrine feedback (75, 245). The proliferative effects of β-endorphin in adult hippocampal neuronal progenitors are mediated through a signaling pathway involving phosphatidylinositol-4,5-bisphosphate 3-kinase (PI3-kinase), [Ca^2+^]_i_, and MAPK activation (245). Importantly, in addition to neurons, the adult hippocampus contains multipotent progenitors that can also give rise to astrocytes and oligodendrocytes (259), so in addition to being mitogenic, β-endorphin can influence the balance of neuronal and glial types in the hippocampus. Adult neural progenitors in the hippocampus can express MOR and DOR, but not KOR (75). Broad-acting opioid receptor antagonists such as naloxone increase the proliferation of neuroblasts, but cause corresponding decreases in the production of astroglia and oligodendroglia *in vitro* (75, 245). Incubating adult rat hippocampal progenitors with the more selective MOR or DOR antagonists, β-funaltrexamine (10 µM) or naltrindole (1 or 10 µM), respectively, for 48 h increases the number of newly generated neurons, while reducing the number of astrocytes and oligodendrocytes in culture (75). This suggests the effects are selectively meditated by MOR or DOR or potentially MOR–DOR heteromeric complexes (260). Additional evidence that MOR activation inhibits adult neurogenesis is provided by findings that deleting MOR increases the generation of new neurons (261). The results intimate that in addition to modulating the proliferation and death of neuronal and glial precursors, opioids may additionally influence the cell fate decisions of bi- or multi-potential neural cell precursors in the SGZ.

## Opioids and Adult Neurogenesis Outside the Hippocampal SGZ

Recently, β-endorphin-expressing neurons in the hypothalamus were discovered to innervate and regulate the division of adult neural progenitors in the anterior ventral VZ–SVZ that will become deep granule interneurons within the olfactory bulb (262). In these studies, the selective KOR agonist ICI 204448 (263) was demonstrated to increase the proliferation of VZ–SVZ progenitors and this was prevented by the selective irreversible KOR antagonist 2-(3,4-dichlorophenyl)-*N*-methyl-*N*-[(1*S*)-1-(3-isothiocyanatophenyl)-2-(1-pyrrolidinyl)ethyl]acetamide hydrochloride (DIPPA) (264). While β-endorphin typically acts as a preferential agonist at MOR and to a lesser extent DOR, it is nevertheless modestly potent at KOR (265). It is interesting that the same *POMC* gene-derived neuropeptide, β-endorphin, affects adult neurogenesis in the hippocampal SGZ through actions at MOR and DOR (75, 245), and in the SVZ associated with the lateral ventricles *via* actions at KOR (262).

### Opioids and Adult NPC Fate Decisions

If the selective MOR antagonist, β-funaltrexamine (10 µM) increases the number of newly generated neurons, while reducing the number of astrocytes (75) (as noted above), does exposure to opiate drugs, most of which are preferential MOR agonists, direct the fate of NPCs toward an astroglial lineage? Xu and coworkers have addressed this question and found that opiates can promote the differentiation of hippocampal NPCs toward an astroglial fate (266). Moreover, these investigators also demonstrate that although both morphine and fentanyl can promote NPCs toward an astroglial fate by activating MORs, they do so by activating different downstream signaling pathways (discussed below) with different consequences (266). By contrast, an escalating dose of methadone, also a preferential MOR agonist and frequently used in maintenance therapy for opiate addiction, failed to alter adult hippocampal neurogenesis (267). The implications of these findings are important and indicate that functional selectivity or biased agonism [reviewed in Ref. (126, 268, 269)] is operative. As noted earlier, this implies that different opioid agonist ligands, acting through MORs, can differentially affect development by uniquely coupling to one or more signaling pathways downstream of MOR. For example, upon stimulation, GPCRs, such as MOR, can subsequently activate one or more downstream effectors including Gα and Gβγ (G-protein subunits), and β-arrestin—each of which can uniquely affect cell function. Therefore, each of the frequently abused opiate drugs (e.g., heroin, fentanyl, and oxycodone), newly synthesized designer drugs, and opiate addiction therapies (e.g., methadone and buprenorphine) may differentially effect the maturation and fate of adult hippocampal neural progenitors within the SGZ. Because the molecular mechanisms underlying how a particular agonist ligand selectively activates one or more signaling pathways downstream of MOR are not well understood, it is challenging to predict how a particular drug will affect development without empirical testing. Finally, because there is evidence that biased agonism similarly dictates the actions of opiates throughout maturation, then key aspects of our understanding of the effects of opiates on neuronal and glial development may need revisiting since a majority of studies to date have used morphine to study maturation.

Although the mechanisms by which opiates direct the maturation and fate of adult NPCs in the SGZ are not fully understood, morphine exposure directs NPCs toward an astroglial fate by increasing microRNA-181a (miR-181a) in NPCs *via* a notch1-dependent pathway (270). MicroRNAs such as miR-181a are short non-coding RNA sequences that bind specific classes of target mRNAs thereby interfering with translation. Since a single microRNA can coordinate the interference of a number of related mRNAs, microRNAs can act as master regulators of translation. Notch1 activation triggers a large number of specific transcriptional processes in a cell-specific manner (271), and in the case of adult NPCs, is necessary for morphine to direct NPCs toward an astroglial fate (266). The selective MOR agonist fentanyl (unlike morphine) acts *via* a pathway involving β-arrestin-dependent extracellular signal-regulated kinase (ERK) activation and inhibition of miR-190 production (266, 272), while morphine triggers protein kinase Cε (PKCε)-dependent activation of both ERK and trans-activation response element RNA-binding protein and increased levels of miR-181 (266); both morphine and fentanyl inhibit calcium/calmodulin-dependent protein kinase type IIα (CamKIIα) (266). Importantly, the actions of both morphine and fentanyl were prevented by the selective MOR antagonist Cys^2^-Tyr^3^-Orn^5^-Pen^7^-amide (CTOP) suggesting morphine’s effects were mediated by specific MORs and that biased agonism is responsible for any differences between morphine and fentanyl (270).

## Concurrent Effects of HIV

We have been particularly interested in the concurrent effects of HIV and opiates on CNS development, as HIV and opiate abuse are linked epidemics, highlighted by outbreaks of HIV in communities suffering from the recent surge in opiate addiction (4). In humans, the period of CNS development extends through adolescence, with full myelination in prefrontal cortex and attainment of executive function not achieved until early in the third decade of life (273–275). Individuals who are vertically infected at birth, or who become HIV+ during the period of CNS development, have a high degree of neuropathology and behavioral deficits (276, 277). We have hypothesized that this is due to specific effects of HIV, HIV proteins, and/or inflammation on developing NPCs, and that concurrent exposure to opiates worsens these outcomes. Our *in vitro* work has shown that the HIV-1 protein Tat, as well as supernatant from HIV-infected cells that contains virions, viral proteins, and inflammatory mediators, can reduce the proliferation of both murine and human neural progenitors, and that concurrent exposure to morphine exacerbates these anti-proliferative effects (278, 279). A similar outcome reported by others showed that Tat-morphine exposure prolonged the Gap 1 (G_1_) phase of the cell cycle, and was dependent on the cyclin-dependent kinase inhibitor p21 (280). In neonatal mice exposed to HIV-1 Tat from embryonic day 17 to postnatal day 7, progenitor proliferation was also reduced, and 4 days of morphine co-exposure further reduced proliferation in SRY-Box 2-immunoreactive (Sox2+) cells (278). Although effects of morphine and Tat were not observed on overall cell populations in the striatum, this may require a longer period of morphine exposure.

**Figure 6:**
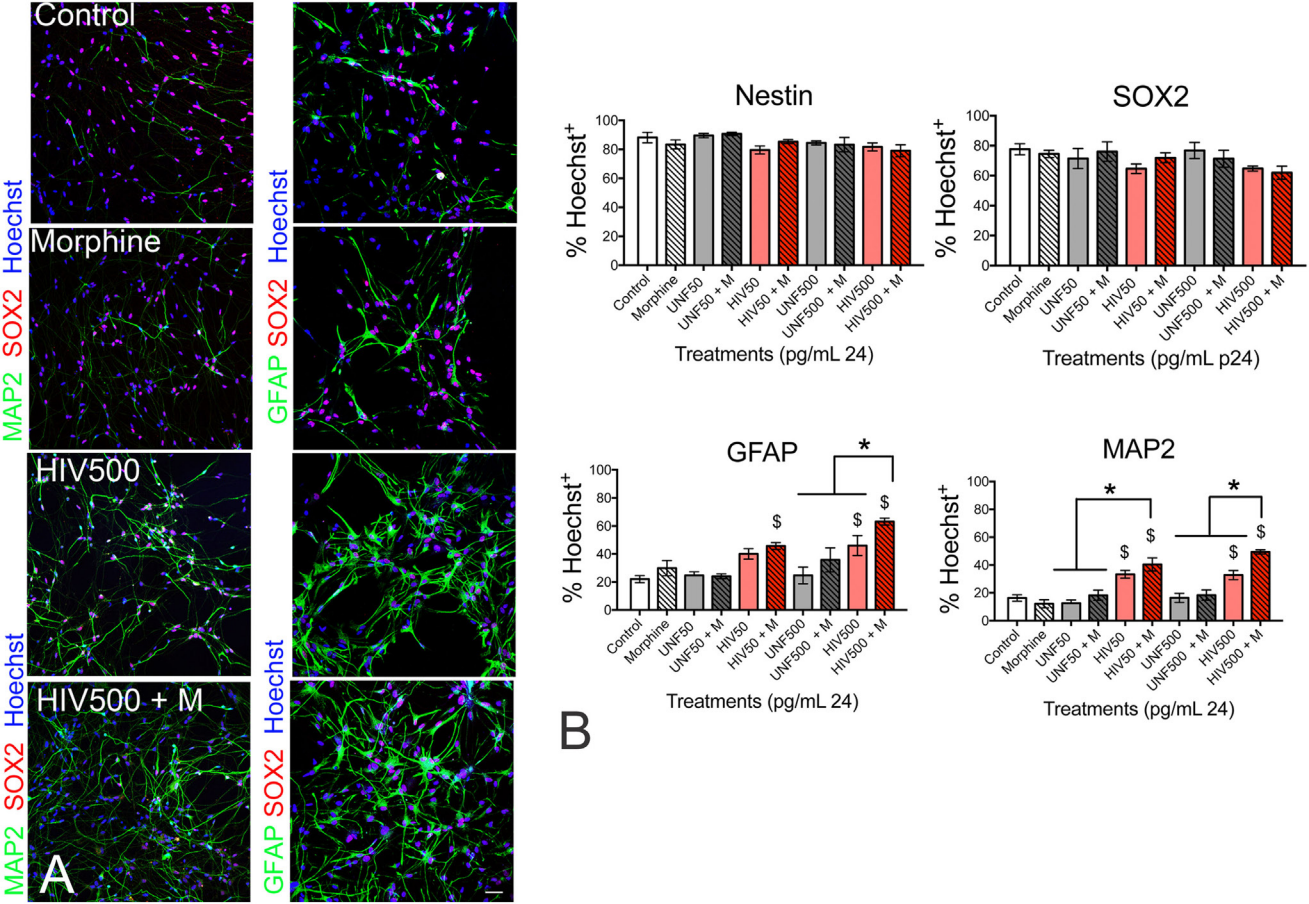
HIV-1 ± morphine co-exposure promotes immature neural progenitor cells (NPCs) to become neurons and astrocytes. **(A)** Representative immunostaining of SRY-Box 2 (SOX2—a marker of undifferentiated NPCs; *red fluorescence*), microtubule-associated protein 2 (MAP2—a neuronal marker; *green fluorescence—left-hand panel*), and glial fibrillary acidic protein (GFAP—an astroglial marker; *green fluorescence—right-hand panel*) in human NPCs treated with morphine (500 nM), spent medium (supernatant) from HIV-1-infected (HIV_sup_) peripheral blood mononuclear cells (PBMCs) containing 500 pg/ml of the HIV-1 p24 capsid protein alone (HIV 500) or with morphine (HIV 500 + M) for 12 days in cell culture. **(B)** Analysis of human NPCs continuously treated with morphine, HIV_sup_ at 50 pg/ml p24 (HIV 50), 500 pg/ml p24 (HIV 500) alone or with morphine (M), and equal dilutions of supernatant from uninfected PBMCs (UNF50 or UNF500) alone or with morphine (M). The percentage of NPCs expressing nestin (transiently expressed by immature neural cells), SOX2, MAP2, and GFAP from each treatment group was calculated from >200 cells per study. Error bars show mean ± SEM from *n* = 5 separate studies each using NPCs derived from independent tissue samples. ^$^*p* < 0.05 versus control; *p < 0.05. Reprinted from Ref. (279); Copyright (2017), with permission from Wolters Kluwer Health, Inc.

Since HIV is a human-specific disease, we re-examined the findings in other models using a human primary neural progenitor system exposed to HIV to more closely mimic aspects of the disease. Human progenitors derived from 8 to 10 week *in utero* tissue samples were exposed to various HIV ± morphine treatment regimens. While morphine treatment over 48 h did not by itself affect human NPC proliferation, morphine did significantly enhance the anti-proliferative effect of HIV exposure acutely, and resulted in an altered doubling time (279). In the 2-week period after the removal of growth factors that sustain the progenitor phenotype, HIV alone accelerated the appearance of both astroglial and neuronal markers in human NPCs, and morphine significantly accelerated this premature differentiation process (Figure 6). As these results were obtained in 8–10-week human gestational tissue, they are especially relevant to developing systems exposed to opiates ± HIV. They mirror the findings discussed earlier in mice exposed perinatally and in murine cultures, which also showed profound, interactive reductions in progenitor proliferation due to morphine-HIV-1 Tat exposure. Overall, these outcomes suggest that chronic exposure of the developing CNS to morphine or HIV, but especially to the combination, is likely to alter the production of mature cells, as well as the differentiation choices of progenitors, biasing the balance of neuronal and glial populations produced as brain regions mature. Although the consequences of these effects have not yet been defined, they are potentially quite damaging to brain function. This study also confirmed that, at least under these conditions, NPCs could be infected and produce new virions. Of great importance, morphine enhanced the production of new HIV virions by naïve human progenitors in a serial dilution and passaging assay. Opiates may thus have the effect of raising viral titers and worsening neurodegenerative outcomes in the developing brain.

## Outstanding Questions/Understudied Areas

Although the main purpose of this review has been to survey prior studies exploring the maturational effects of opiates on neurons and glia, it seems appropriate to briefly suggest new directions for future research in this area.

### To What Extent Do Developing Cells Adapt to Chronic Opiate Exposure?

This review has emphasized the decline in cellular growth observed with relatively acute, constant, opiate exposure. However, outside of controlled, clinical settings opiate exposure is rarely acute, and blood levels of opiates often fluctuate widely within the same individual. Tolerance and dependence can be observed at the cellular level as evidenced by dichotomous regulation of adenylate cyclase responsiveness in NG108-15 neuroblastoma × glioma cells following 1–4 days exposure to morphine (10 µM) and after morphine withdrawal (281). However, limited studies have examined the cellular consequences of sustained opioid exposure in developing neurons or glia. Even fewer studies have examined the consequences of opioid tolerance or withdrawal on cellular maturation. Although the well-documented decline in growth after relatively short opiate exposure times has been emphasized in this review, a return to near normal numbers of immature astrocytes despite sustained exposure to the preferential DOR agonists Met-enkephalin (171) or DPDPE (169) has been observed and suggests that compensatory factors can become operative. There is a paucity of literature examining the neurodevelopmental consequences of opiate tolerance or withdrawal on neuronal and glial maturation in the developing brain *in vivo*. An understanding of the cellular mechanisms underlying adaptive responses to chronic opiate exposure is crucial toward understanding the consequences of perinatal opiate exposure.

### Modeling the Pharmacology of Self-Administration during Maturation

An even greater challenge is to better model the pharmacology of opiate drug exposure as seen with opiate self-administration in addicts during perinatal/postnatal development. The pharmacokinetics of “on-off” (intermittent, including contingent and non-contingent patterns of administration) versus “steady state” (continuous, e.g., as seen with an osmotic minipump or time-release drug implant) drug administration (282) and resultant pharmacodynamic differences are likely to have a significant impact on neuronal and glial maturation. Addicts inject opiates repeatedly, 3–4 times per day, to maximize the rewarding effects of the drug. Fluctuating levels of opiates are responsible for a relative “high” or “rush” and 3–4 periods of relative/mild withdrawal per day disrupt a wide variety of physiological systems and are through to be inherently more destabilizing than exposure to constant drug levels (282, 283). We speculate that fluctuating levels of opiates are more likely to have deleterious effects on CNS maturation than exposure to constant drug levels, which infers that the amount, frequency, and duration of drug exposure are important determinants of developmental outcome. Intermittent and fluctuating levels of opiates would be encountered during *in utero* exposure of offspring of opiate addicts; more constant levels of opiate exposure would be encountered when opiates were provided to neonates and newborns for pain management (284). Self-administration studies are particularly challenging to perform in pregnant or lactating dams, and may not be possible in young animals. Although the neuropharmacological and neurobehavioral consequences of intermittent versus continuous drug exposure, as well as contingent versus non-contingent opiate administration, have been examined in detail in adult animal models (285), the consequences of differing patterns of opiate administration on CNS maturation in children are unclear.

### What Are the Long-Term Consequences of Opioids on Development?

A vast majority of studies in animals involve relatively acute exposure to opioids with durations lasting 1 week or less; with notable exceptions (255), studies that extend opiate exposure durations beyond 2 weeks are rare. In this regard, the clinical literature may provide more insight than studies examining acute bouts of opiate exposure in animals—especially in rodents in which development is relatively rapid. Moreover, a greater understanding of the pharmacokinetic consequences of opiate exposure (as noted in the preceding paragraph), as well as potential differences among opiate treatment options such as methadone versus buprenorphine (286, 287), are critical for designing strategies for treating mothers or offspring with opiates when medically necessary. Drug exposure causes long-term changes in neuroplasticity during the switch from casual drug use to an addicted state (17, 136, 288). Opiate-dependent alterations in neural circuitry may be greater during maturation when increased levels of MOR expression are evident in rodents (discussed earlier) and humans (289), and large-scale changes in CNS organization and synaptogenesis are occurring throughout the brain. For example, infant rats made briefly tolerant and dependent to fentanyl from P14 to P17, but not exposed thereafter, show lasting tolerance to morphine as juveniles and young adults 1 year later (290). By contrast, it cannot be assumed *a priori* that opiate exposure during development will only effect neural circuitry involved in addiction. In fact, this is unlikely the case since in many instances the cellular targets, and regional patterns of opioid peptide and receptor expression, during maturation are transient (and not present in adults) or thought to be unrelated to addiction (34, 50, 153, 291–293). It seems plausible that exposure to opiate drugs at critical periods during development results in lasting or permanent alterations in the structure and function of neural circuits directly related, as well as unrelated, to addiction.

## Author Contributions

KH and PK both wrote the article and edited the submitted version.

## Conflict of Interest Statement

The authors declare that the research was conducted in the absence of any commercial or financial relationships that could be construed as a potential conflict of interest.
